# Editorial: Kidney transplantation and immune-mediated nephropathies, Volume II

**DOI:** 10.3389/fimmu.2022.1009318

**Published:** 2022-08-23

**Authors:** Josep M. Grinyó, Vladimir Tesar, Piergiorgio Messa

**Affiliations:** ^1^ Department of Clinical Sciences, University of Barcelona, Barcelona, Spain; ^2^ Department of Nephrology, General University Hospital, Charles University, Prague, Czechia; ^3^ Unit Nephrology Dialysis & Renal Transplantation, Policlinico Milano, Milano, Italy

**Keywords:** kidney transplantation, IgA nephropathy, IgA vasculitis, systemic sclerosis, antibody mediated rejection

Kidney transplantation is a quite unique setting in which allo and autoimmune-mediated mechanisms may induce renal parenchymal injury along the entire life of the renal allograft. Autoimmune nephropathies are leading entities for the development of end-stage renal disease (ESRD) and their recurrence after transplantation accounts for an important proportion of renal graft losses in the long term (1). Moreover, rejection in its different forms, may also add a detrimental effect on recurrent autoimmune nephropathies contributing to graft loss.

IgA nephropathy (IgAN) is one of the most prevalent glomerulonephritis in Europe and Asia and frequently lead patients to dialysis and transplantation. In this second volume of Kidney Transplantation and Immune-Mediated Nephropaties, Maixnerova et al. report a retrospective study on the outcome of IgAN after renal transplantation in Czechia. The study integrates the assessment of risk factors before and after transplantation on renal outcomes, including baseline histology leading to the diagnosis of IgAN as the cause of ESRD. This cohort with 313 patients may result representative on the evolution of IgAN in Central Europe. The reported incidence of IgAN recurrence is 14%, rather low, as reported by the authors. This finding may be derived from the diagnostic tool used for establishing the recurrence. In the study, it is reported that all patients had protocol biopsies at 3 months after transplantation, which may be soon to identify histological recurrence. It might be interesting to have information on the proportion of patients with an initially negative protocol biopsy for IgAN recurrence with a subsequent positive for cause biopsy. This work describes several important findings such as that the faster the progression from the initial diagnostic renal biopsy in native kidney to ESRD, the shorter the time from transplantation to graft failure (11.2 vs 6.1 years). Importantly, in this cohort the main cause for graft failure was IgAN recurrence which was associated with 50% of graft loss. This study also identifies early microscopic haematuria and proteinuria as risk factors for graft failure. Interestingly, erythrocyturia worsens graft prognosis in cases of histologically proven recurrence. Antibody-mediated rejection, recipients’ age, and age at the time of renal biopsy are also detrimental factors for graft survival.

Song et al. review the pathogenesis of another IgA-related disease such as IgA vasculitis (IgAV), formerly known as Henoch-Shönlein purpura, a leukocytoclastic vasculitis, which may affect several tissues and organs. Similarly to IgAN, the main characterized abnormality in the pathogenesis of IgAV has been the identification of galactose-deficient IgA1 (Gd-IgA1). The production of aberrant IgA and anti-Gd-IgA1 autoantibodies, the formation of immunocomplexes and tissue deposition, which triggers inflammation and organ injury, integrates a comprehensive multi-hit model in the pathogenesis of IgAV. The production of autoantibodies might be induced by the residues in Gd-IgA1 or mucosal antigens with a similar structure of Gd-IgA1. Variations in the expression or activities of enzymes that catalyze O-glycosilation of IgA1 in B-cells may account for the deficiency of galactose of IgA1. Among other players, the deposition of immunocomplexes in the kidney may be favoured by the increased expression in mesangial cells of transferrin receptor, which is a IgA1 receptor. Such abnormalities persist after renal transplantation and may contribute to the recurrence of IgAV in the renal allograft which is associated with a risk of graft loss ranging from 7.5% to 13.6% at 10 years after transplantation (2, 3).

In a narrative review, Mariati et al. update the information on Systemic Sclerosis (SSc) emphasizing the renal involvement, renal replacement therapy (RRT), and especially renal transplantation (RT). The bad prognosis of SSc improved with the introduction of Angiotensin-converting enzyme inhibitors (ACEi) 40 years ago which allowed a better control of severe hypertension, and renal function recovery in a relatively important proportion of patients even after the initiation of RRT. As described in the review, the most severe renal complication of SSc is scleroderma renal crisis (SRC). This SSc complication approximately occurs in a 20% of patients, and it was found that 80% of patients under RRT had developed ESRD secondary to SRC. Recent data indicate that in patients SRC, anti-RNA polymerase III antibodies are frequently detected. Data from 3 registries on patients treated with RRT show reduced survivals in SSc (30% approximately at 5 years) in comparison with patients with ESRD secondary to other nephropathies. In selected candidates, RT is the best therapeutic option as it may offer high survival rates and an improved quality of life. The indication for RT in patients with ESRD secondary to SSc may be delayed after the initiation of RRT to reduce the risk of early disease relapse. The authors report that according to registries’ data the identified recurrence rates are rather low and that graft survivals are comparable to other renal diseases, which supports RT as an important therapeutic option. Despite the use of distinct immunosuppressants in the treatment of SSc, there are no specific immunosuppressive regimens recommended for induction and maintenance immunosuppression after transplantation in patients with SSc.

One of the main causes of renal allograft loss is active antibody mediated rejection (aABMR) and chronic active ABMR (caABMR) results in permanent graft attrition in mid and long-term after transplantation. No highly effective therapy has been developed so far for aABMR. Cabezas et al. review the efficacy and safety of the use of Tocilizumab (TCZ), an IL-6 receptor (IL-6R) monoclonal antibody, in the treatment of aABMR,. TCZ has successfully used in the treatment of inflammatory conditions and autoimmune diseases. The physiologic effects of IL-6 on the regulation of inflammation, development and maturation of T and B and plasma cells, involved in the pathophysiology of aABMR provide the mechanistic rationale for the use of TCZ in this type of rejection. Six non-randomized reviewed studies with low numbers of patients on the use of TCZ in the rescue treatment of caABMR (3), first-line treatment of caABMR (2) and one study in the rescue therapy of aABMR show promising results, with an adequate efficacy/safety balance, although with heterogeneous outcomes probably due to the distinct baseline characteristics of patients. The interesting findings on the reduction of microvascular inflammation and decrease in donor-specific antibodies support the mechanistic properties of TCZ. The role of targeting the IL-6/IL-6R axis in ABMR is addressed in an ongoing phase 3 randomized clinical trial (ClinicalTrials.gov. NCT03744910).

## Author contributions

All authors listed have made a substantial, direct, and intellectual contribution to the work and approved it for publication.

## Conflict of interest

The authors declare that the research was conducted in the absence of any commercial or financial relationships that could be construed as a potential conflict of interest.

## Publisher’s note

All claims expressed in this article are solely those of the authors and do not necessarily represent those of their affiliated organizations, or those of the publisher, the editors and the reviewers. Any product that may be evaluated in this article, or claim that may be made by its manufacturer, is not guaranteed or endorsed by the publisher.
